# Immune modulatory oncolytic virus for osteosarcoma therapy

**DOI:** 10.21203/rs.3.rs-9488970/v1

**Published:** 2026-05-04

**Authors:** Sumbul Khan, Theresa A Higgins, Isabella Shimko-Lofano, Maninder Sandey, Emma Logsdon, Gracie Bunch, Yasin Fatemi, Leah MK Hoffman, Bruce F Smith, Payal Agarwal

**Affiliations:** 1 Scott Ritchey Research Center, College of Veterinary Medicine, Auburn University, Auburn, AL; 2 Department of Pathobiology, College of Veterinary Medicine, Auburn University, Auburn, AL; 3 Department of Mathematics and Statistics, College of Sciences and Mathematics, Auburn University, Auburn, AL; 4 Department of Industrial and Systems Engineering, Samuel Ginn College of Engineering, Auburn University, Auburn, AL

## Abstract

Osteosarcoma (OS) is a highly aggressive bone malignancy that predominantly affects children and young adults. There have been no major changes in patient survival in the last four decades. Therefore, new therapeutic interventions are needed. We have developed an enhanced conditionally replicative canine oncolytic adenovirus, CAV2-AU-M3, armed with an anti-PD1 heavy chain antibody (HcAb), and evaluated its efficacy against osteosarcoma across four canine cell lines. CAV2-AU-M3 was characterized for its infectivity, lytic properties, and anti-PD1 Ab production in monolayer and spheroid cultures. Additionally, the impact of intra-tumoral administration of the virus on tumor growth was measured in a mouse model. Our study demonstrates that CAV2-AU-M3 infects and lyses different canine OS cell lines at varying rates but produces anti-PD1 Ab at similar levels across all osteosarcoma cell lines. Additionally, anti-PD1 Ab produced by CAV2-AU-M3 can effectively bind the cell-surface PD1 receptor and inhibit the binding of PDL1 to PD1 receptors.

## INTRODUCTION

Osteosarcoma (OS) is a malignant bone tumor that most commonly occurs in children and adolescents (3–5% of childhood cancers) (1–3). OS predominantly occurs in long bones, with higher frequency near the knees, hips, and shoulders (4, 5). Despite multidisciplinary treatment, including surgery, chemotherapy, targeted therapy, radiation therapy, and even combination therapies, the survival rates for patients with OS have not improved in the last four decades, especially for patients with metastatic disease (6–11). The 5-year survival rate is less than 20% in metastatic patients (8, 10)

Cancer immunotherapy stimulates the host’s own immune system against the tumor and turns the immunologically ‘cold’ tumor microenvironment (TME) into ‘hot’. Immunotherapies provide long-term defense against tumors and enable the immune system to recognize and target tumor cells in the event of recurrence or metastasis. Immunotherapies, including immune checkpoint inhibitors (ICIs), genetically modified T cells, immune modulators, and cytokines, have shown remarkable improvements in cancer treatment in *in vivo* models with fewer side effects, including OS (12–14). Immune checkpoints, such as PD1/PDL1 interaction, cause immune evasion in the tumor microenvironment by inactivating cytotoxic T cells (12–14); and blocking this interaction leads to tumor growth inhibition, Treg decrease, and increased tumor-infiltrating lymphocytes *in vitro* and *in vivo* mice OS model (14). However, systemic delivery of anti-PD1 mAb can cause immune-related adverse effects (15) in organs such as the liver, lungs, kidneys, and heart (16). It is also possible that systemic anti-PD1 mAb may not reach the solid tumor site due to the tumor stroma (17).

**Figure 1:**
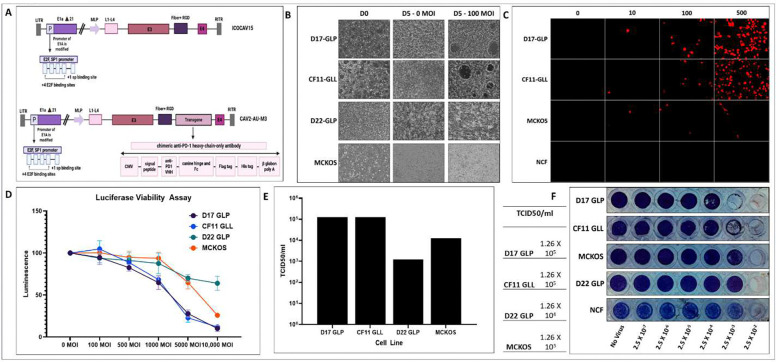
CAV2-AU-M3’s infectious and cytolytic properties in canine osteosarcoma cells in 2D culture. (A) Graphical Representation of ICOCAV15 and CAV2-AU-M3. (B) Cytopathic effects of CAV2-AU-M3 at 100 MOI in Canine OS cell lines (D17 GLP, CF11 GLL, D22 GLP, and MC-KOS) visualized using inverted fluorescent microscopy (Keyence) at day 5 post-infection at 10× magnification. (C) Immunofluorescence using mouse anti-CAV2 (VMRD) primary antibody and anti-mouse IgG Texas-red secondary antibody was performed in D17 GLP, CF11 GLL, and MCKOS cell lines and primary NCF cells at 0, 10, 100, and 500 MOIs. Images were captured using a Keyence microscope at 10X 48 hrs post-infection. (D) Luciferase assay for cell viability in OS cell lines at 100, 500, 1000, 5000, and 10000 MOIs at day 5 post infection. (E) TCID50/ml values of CAV2-AU-M3 in OS cell lines. (F) Tabular representation of the data from (E). (G) Crystal violet assay to demonstrate TCID50/ml values in all OS cell lines. Each row depicts the virus dilutions.

To address these issues, we have developed a conditionally replicative oncolytic canine adenovirus type 2 vector (CAV2-AU-M3; Figure 1A) armed with a secretory chimeric anti-PD1 heavy-chain-only antibody. Conditionally replicative oncolytic adenoviruses (CRAds) lyse tumor cells and induce systemic anti-viral immunity (DAMPs and cytokine secretion from tumor cells), along with anti-tumor immunity (18–21). Additionally, oncolytic virus therapy improves anti-PD1 immunotherapy by promoting intratumoral cell infiltration (22) and localized secretion of anti-PD1 Ab to avoid adverse effects (23). CAV2-AU-M3 is designed to result in local, sustained production and secretion of anti-PD1 Ab into the TME to avoid systemic exposure and cross the tumor stromal barrier.

Previously, we designed a similar oncolytic virus (CAV2-AU-M2) that encoded anti-PD1 VHH, which was released only after cell lysis. CAV2-AU-M3 encodes and secretes the chimeric anti-PD1 Ab, which is more stable due to the presence of the canine Fc region in the antibody (24). In this study, we have characterized the CAV2-AU-M3 virus, its cytotoxic properties, and its ability to produce and secrete anti-PD1 Ab.

## Material and Methods

### Cell lines:

Canine osteosarcoma cell lines (D17, CF11, and D22) were purchased from ATCC. The additional canine osteosarcona cell line MCKOS and the hunan packaging cells HEK293 17SF were kind gifts fron Dr. Rinas J. Orentas (Miltenyi). The Human HEK293-K9 PD1 (expressing the canine PD1 receptor on the cell surface) cell line was a generous gift from Dr. Maninder Sandey. Canine normal fibroblast cells (NCF) were isolated and cultured from a donor dog’s subcutaneous fat tissue. All cell lines and their respective clones were cultured according to standard lab procedures (25).

### Adenovirus and Lentivirus vector construction and packaging:

Genetically modified adenoviruses CAV2-AU-M3 (CAV2-E1AΔ21-RGD-sec.anti-PD1; Figure 1A) were generated fron the plCOCAV15 plasnid as a backbone using CRlSPR/Cas9 technology, as published previously (26). The anti-PD1 HcAb was developed by conjugating the anti-PD1 sdAb (25) to canine Fc and adding a secretory signal. The sequence of secretory anti-PD1 Fc conjugated antibody includes FLAG and His-tags at the C-terminus of the sequence. We chose the sdAb clone with the highest binding affinity for canine PD1 (APD-1D5). Anti-PD1 HcAb sequence was then inserted under the control of the CMV promoter between the fiber and E4 sequences (26).

GFP/ffluc lentivirus was produced using the pALD-Lenti system (Aldevron), employing the pGFP/ffluc plasmid and packaging plasmids. Lentivirus packaging plasmid, pALD-Rev, pALD-gagpol, and pALD-VSV-G were purchased from Aldevron. pGFP/ffluc was a kind gift from Dr. Rimas J Orentas (Miltenyi). pGFP/ffluc, along with packaging plasmids, were transfected into HEK293/17SF cells using PEIpro (Polyplus) transfection system. Sodium butyrate (8mg/ml) was added 24hrs post transfection. On day 4, cells were harvested and centrifuged at 2000rpm for 10 min at room temp. The supernatant was collected and filtered through a 0.45 μm filter, then centrifuged at 10,000 × g for 4 hours with slow acceleration and a brake at 4°C. The pellet,post centrifugation, was suspended in 400ul serum-free media and stored in small aliquots at −80°C.

For lentivirus titration, canine OSW cells were cultured and transduced with GFP/ffluc lentivirus at different concentrations. The transduced cells expressing GFP were quantified using flow cytometry. Titer was calculated using the following formula:

$$\text{Lentivirus}\;\text{Titer}\;\text{(TU/mL)=}\frac{\text{Number}\;\text{of}\;\text{Cells}\;\text{at}\;\text{Time}\;\text{of}\;\text{Transduction}\;(100,000)\;\times \;(\%\;\text{of}\;\text{Positive}\;\text{Cells/100})}{\text{Volume}\;\text{of}\;\text{Undiluted}\;\text{Virus}\;\text{Added}\;(\text{mL})}$$

### Generation of GFP+ osteosarcoma cell lines:

Canine osteosarcoma cell lines D17, CF11, and D22 were transduced with GFP/ffluc lentivirus (MOI 1). Five days post-infection, GFP-positive cells were sorted (Cyto FLEX, Beckmann) into 96-well plates at a density of one cell per well. Plates were monitored daily for growth and GFP expression. Selected clones from the 96 well plates were expanded gradually to T75 flasks. GFP expression was confirmed via fluorescent microscopy on a Keyence BZ-X800 microscope. Luciferase expression was confirmed via SteadyGlo luciferase assay (Promega cat #E2510) as per the manufacturer’s instructions. D17-GLP, CF11-GLL, and D22-GLP clones were selected to perform further experiments.

### CAV2-AU-M3 viral infections:

#### 2D (monolayer) cell culture:

Canine osteosarcoma cell lines, D17-GLP, CF11-GLL, MCKOS, and D22-GLP, were cultured in a 12-well plate overnight. On the ne:t day, nedia was renoved and cells were washed once with 1X PBS (Phosphate Buffer Saline, Corning: cat#21–040-CV). Cells were infected with CAV2-AU-M3 at nultiple nultiplicities of infection (MOl; 0, 10, 100, 500, 1000, 5000, and 10,000) in low-serun nediun (500 ul DMEM, 2% FBS). After 2 hrs of incubation at 37°C, 500 ul of additional DMEM (5% FBS) was added. lnfected cells were naintained in DMEM (5% FBS), and the nedia was changed on days 3 and 5. The Keyence BZ-X800 inaging systen was used to capture bright phase inages on days 0, 3, and 5.

#### 3D (spheroid) cell culture:

Spheroids were forned by plating 5000 canine osteosarcona or NCF cells on a low attachnent round-botton 96- well plate (Corning ref #7007) in 50 ul of conplete nedia and 50 ul of e:tracellular natri: (ECM) (30 μg/nL; Collagen l, Therno #5056–20ML). The plates were centrifuged at 500 g for 5 nin and incubated at 37°C for 72 hrs with regular nonitoring of spheroid fornation. lnfections were perforned at 72 h using CAV2-AU-M3 (MOl: 0, 100, and 1000). Partial nedia changes were preforned and inages were captured (Keyence BZ-X800 inaging systen) on day 0, 4, 7, 10, and 13. The dianeters of the spheroid inages were calculated using lnageJ. The calculated spheroid area on days 7 and 10 was nornalized to the spheroid size of Day 0.

#### In vivo nodel:

D17-GLP 5X10^6^ cells in 0.2 nl liquid Matrigel (Corning ref#354230) were subcutaneously injected into the left flanks of 6-week-old NOD. Cg-Prkdc^scid^ Il2rg^tm1Wjl^/SzJ fenale nice. Mice were randonly divided into two groups of 5 each. When the tunor size reached 100 nn^3, group 1 (n=5) and group 2 (n=5) were treated with 1×10^9 CAV2-AU-M3 virus particles and 1X PBS in a volune of 50ul. Virus/1X PBS was injected intratunorally in the tunors. The status of the nice and tunor size were nonitored daily during the 3 days after virus adninistrationand then 3 tines a week for XX weeks. Tunor volune was defined by the equation V(mm3)=(W2×L)/2, where W and L are the width and the length of the tunor, respectively. When aninals fron each group displayed uncontrolled tunor growth, the nice were euthanized.

### Spheroid histology:

Spheroids were infected with 0, 100, and 1000 MOls of CAV2-AU-M3 and cultured for 13 days. On day 13, spheroids were collected, washed with 1 X PBS, and fi:ed in 4% PFA (Parafornaldehyde) at roon tenperature for 42 nin. For better visualization of the spheroids, they were pre-stained with erythosine B (0.2%) (Signa cat#200964–5G) in 1X PBS and incubated for 3 ninutes at roon tenperature. Spheroids were washed 3 tines with 1X PBS and deposited in a cryonold containing liquid HistoGel (Therno cat#HG10144). The gel was cured on an ice block for 10 ninutes. The encased spheroids were subsequently transferred to nesh cassettes, and placed in 70% ethanol until processing. Sanples were processed using the routine cycle progran in a Leica TP1040 processor and enbedded in Leica Paraplast X-tra paraffin using a Sakura Tissue Tek lV enbedding station. The blocks were sectioned at 4 μn using a Leica 2125RM nicrotone, with sections placed on Matsunani APS-charged slides (Cancer Diagnostics cat#SUAPS11). Slides were air-dried at room temperature overnight. Prior to staining, the slides were “baked” in an incubator at 60°C for 10 minutes. Slides were manually stained using Cancer Diagnostics (CDI) Gill’s II henato:ylin (cat#CM5951) and Eosin phlo:ine B (cat#EM00PG) working solutions. Slides were coverslipped with #1 24–50nn slips using Richard Allen CytoSeal XYL.

### Innunofluorescence (IF):

#### 2D:

Cells were plated at 50,000 cells per well in eight-chanbered slides (Corning) and cultured overnight at 37 °C. Slides were infected with 0, 10, 100, and 500 MOl as per the 2D infection protocol above. 48hrs post-infection, slides were washed with PBS and fi:ed for 20 nin in 4% PFA. Slides were then washed 1: with PBS and incubated in block buffer (10% nornal horse serun (Vector Laboratories cat #S-2000–20), 0.4% triton, and 1X PBS) for 1hr at roon tenperature. Prinary antibody (Mouse anti-CAV2: VMRD; rabbit anti-VHH-iF647: GenScript cat #A01994–200) incubation was perforned for 1 hour at roon tenperature in 1X wash buffer (1% nornal horse serun, 0.4% Triton X-100 in 1X PBS). Slides were washed 1–2: with PBS. Slides were incubated with secondary antibody (Te:as-red goat anti-nouse lgG lgG: lnvitrogen ref #T6390) for 1 hour at roon tenperature. The slides were washed 1–2: with PBS, the slide chanbers were renoved, and the slides were nounted with DAPl nounting nediun (Vector Laboratories; cat# H-1800-Z). Slides were inaged on the Keyence BZ-X800 nicroscope.

#### 3D:

Slides were incubated at 60 °C for 10 min. Slides were deparaffinized and rehydrated with the following washes for 5min each: xylene x3, 100% ethanol x2, 90% ethanol, 75% ethanol, 50% ethanol, and distilled water. After washing, slides were transferred to 10 mM sodium citrate and boiled in an InstaPot for 10 minutes on the sterilization cycle. After completion, the slides were incubated in 10 mM sodium citrate at room temperature for 30 min. Slides were then washed in distilled water for 5 min. A PAP pen (Vector Laboratories) was used to draw a wide circle around the periphery of the tissue sample without allowing the sample to dry. Slides were incubated for 15 min at room temperature in wash buffer (1% normal horse serum, 0.4% triton x100, in 1X PBS) and blocked using block buffer (10% normal horse serum, 0.4% triton 100X in 1X PBS) for 1 hour at room temperature in a humid chamber and at 4 °C until the end of the day. At the end of the day, 1:500 primary antibody was added and incubated overnight at 4 °C in a humid chamber. The following day, slides were washed 3x with wash buffer for 10 min each wash. Secondary incubation was done for 1 hour in a humid chamber at room temperature. Slides were washed 1x with 1X PBS for 10 min and mounted with DAPI mounting medium. Slides were imaged on the Keyence BZ-X800 microscope.

### PBMC isolation and T cell activation:

Peripheral blood was collected from healthy donor dogs in 10 mL monoject EDTA (K3) 0.10mL 15.0% solution tubes (Covidien cat#8881311743). Blood was diluted with 25 mL wash buffer (PBS + 2% FBS + 0.5% EDTA) per 10 mL of blood. 35 mL of diluted blood was then layered onto 15 mL of lymphocyte separation medium (Corning cat#25–072-CV) in a SepMate tube (StemCell cat#15450) and centrifuged at 1200 × g for 10min as per the manufacturer’s instructions. The top layer was collected in a 50 mL conical tube, and wash buffer was added to a final volume of 50 mL and then centrifuged at 200 × g for 10 min at 50% acceleration and 0% deceleration to remove platelets. The supernatant was removed, and the pellet was washed twice with 10 mL of wash buffer and centrifuged at 500 × g for 10 min. To remove remaining red blood cells (RBCs), the cell pellet was resuspended in 3 mL 10% RBC lysis buffer (Invitrogen cat#00–4300-54) in sterile water and incubated at 4 °C for 4 minutes. 12 mL of wash buffer was then added to stop lysis, and the cells were centrifuged at 500 × g for 10 min. If visible RBCs remained, this step was repeated. Cell pellet was then resuspended in 10 mL of wash buffer and centrifuged at 500 × g for 10 min.

For T cell purification, the cell pellet was resuspended in 80 μL MACS buffer (PBS + 0.5% FBS + 0.4% EDTA; kept cold during processing) per 10 million cells. Then 10 μL of dog gamma globulins (Jackson cat#004-000-002) were added per 10 million cells, and the mixture was incubated on a rotator for 15 min at room temperature. Next, primary antibodies to CD11b (Biorad cat#MC1777S) 5 uL per 10 million cells, CD11c (Biorad cat#MC1778S) 5 uL per 10 million cells, CD21 (Biorad cat#MCA1781R) 1 uL per 10 million cells, and CD14 (Biorad cat#MCA1568GA) 1 uL per 10 million cells were added and incubated on a rotator for 30 min at 4 °C. Cells were then washed with 2 mL MACS per 10 million cells and centrifuged at 500 × g for 10 min at 4°C. The cell pellet was then resuspended in 80 μL MACS per 10 million cells. Next, 20 μL anti-mouse IgG (Miltenyi cat#130-048-401) beads per 10 million cells were added and incubated on a rotator for 15 min at 4 °C. Cells were then washed twice with 2 mL MACS per 10 million cells and centrifuged at 500g for 10 min at 4 °C. An LS column (Miltenyi cat# 130-042-401) was attached to the magnet (Miltenyi; midi MACS multistand) and rinsed with 3 mL of MACS. The cell pellet was resuspended with 50 μL MACS per 10 million cells and ran through the LS column. The column was rinsed with 500 μL MACS. Purified T cells were diluted to 10 mL in MACS and counted. 100,000 cells were set aside for flow cytometry. Cells were then centrifuged at 500 × g for 10 min at 4 °C. To confirm T cell purity, flow cytometry using anti-CD5 antibody (Invitrogen cat#25-0250-42) was performed.

For activation, purified T cells were cultured in a 6-well plate coated with anti-dog CD3 (Biorad cat#MCA1774GA) and anti-dog CD28 clone 5B8 (generously gifted by Dr. Maninder Sandey). Activation was confirmed after 72 hrs by flow cytometry with anti-dog CD25 (Invitrogen cat#25-0250-42) primary antibody. Activated T cells were then maintained at 1 million T cells per mL in IL-2-supplemented T cell media (TCM; IMDM (Gibco) 10% FBS (Sigma cat#16000-044), penicillin (100 IU/ml, Corning cat#30-002-CI), streptomycin (100 μg/ml, Corning cat#30-002-CI), 1% non-essential amino acids (Corning cat#25-025-CI), and 1% sodium pyruvate (Gibco cat#113600-070) for 30 min for ten days, with media supplemented every 2–3 days and cell counts every 3–4 days.

### Luciferase Cytotoxicity Assay:

Cell cytotoxicity post virus-infection in 2D culture was quantified using Luciferase cytotoxicity assay (Promega). Canine osteosarcoma cell lines D17-GLP, CF11-GLL, MCKOS, and D22-GLP were cultured in a 96-well plate (10,000 cells/well). CAV2-AU-M3 infections were performed as described above. On days 0, 3, and 5, Steady-Glo^®^ Luciferase Assay reagent (Promega cat #E2510) was added in equal amounts of media as per the manufacturer’s directions. Cells and luciferase reagent were incubated at room temperature for up to 30 minutes. Cell lysis was monitored by luminescence using a microplate reader (BioTek Synergy H1). The media was changed before adding the assay reagent on each day.

### TCID50/ml:

All four OS cell lines and NCF were plated in a 96 well plate (10,000 cells/well). The next day, each well was infected with 10-fold dilutions of virus with a starting inoculum of 2.5ul in the top row. After 5 days of infection, crystal violet staining was performed as described previously (27). TCID50 was calculated using the Reed and Munich assay.

### CAV2-AU-M3 Infections and Nickel Colunn Purifications:

CAV2-AU-M3 infections and anti-PD1 Ab purification were done as per our standard lab techniques (25). Briefly, D17-GLP, CF11-GLL, MCKOS, and D22-GLP cell lines were cultured in T75 culture flasks up to 70–80% of confluence. The cells were infected with CAV2-AU-M3 at 100 MOI in DMEM with 5% FBS. 72 hrs post-infection, media and cell lysate were collected. For cell lysate, cells were collected and washed with 1X PBS, then lysed with RlPA buffer (Therno ref #89900) supplenented with 1% Halt Protease lnhibitor Cocktail (Therno). Cells were frozen at −80°C and centrifuged at 14,000 X g. Supernatant was collected and stored at −20°C. Cell culture media was concentrated using protein concentrators (Thermo ref #88528; 10K MWCO). His-tagged anti-PD1 Ab was purified using Ni-NTA spin Kit (Qiagen) as per the manufacturer’s protocol. The purified sanples were stored at −20°C.

### Western Blot:

#### Anti-PD1 Ab identification:

Purified anti-PD1 Ab sanples were boiled for 10 ninutes in Lane narker reducing buffer ((Therno: cat#39000)) and loaded onto NuPAGE 4–12% Bis-Tris gel (Therno ref #NP0323BOX). The chaneleon Duo Pre-stained ladder (Licor ref #928-60000) was used for nolecular weight control. SDS-PAGE Electrophoresis was perforned using 1X NuPAGE MOPS SDS running buffer (Therno #ref B0001) for ~45 nin at 210 Volts. Protein transfer on PVDF nenbrane post-electrophoresis was perforned using 1X Bolt transfer buffer (Therno ref #BT00061) at 20V for 1 hr. The nenbrane was blocked for 2 hrs at roon tenperature using block buffer (1x PBS, 0.1% Tween20, 5% non-fat nilk powder). lncubation with prinary antibody was perforned overnight at 4°C. The nenbrane was washed the ne:t day 3 tines using wash buffer (1x PBS and 0.1% Tween20) for 20 nin each and incubated with secondary antibody for 1 hr at roon tenperature. The nenbrane was washed 3 tines using the wash buffer after secondary antibody incubation. The nenbrane was scanned using LlCOR Odyssey systen. Mouse anti-His-Tag (Therno ref #MA1-21315) and rabbit anti-VHH-HRP (GenScript cat #A01861-200) prinary antibody and lRDye 800 CW goat anti-nouse lgG (LicorBio ref #926-32210) were used. Purified anti-PD-1-D5 sdAb (~15 kDa) was used as a positive control (a generous gift from Dr. Maninder Sandey).

### Flow cytometry:

#### PD1 Binding:

Human HEK293-K9PD1 cell line cells and T cells were blocked for 1 hr at room temperature using blocking buffer (FACS + 10% nornal nouse serun; lnnunoReagents cat #SP-002-VX5). Cells were labelled with prinary antibody (purified anti-PD1 Ab fron each cell line and NCF cells) for 1 hr at roon tenperature post-blocking. Unlabeled cells were used as negative control. Cells were washed with FACS buffer (1X PBS + 1% B.S.A.; bovine serun albunin; VWR cat #0332, + 200nM EDTA, Therno ref #15575–038) and labelled with nouse anti-His-Tag APC-conjugated secondary antibody (R&D: cat #lC050A) for 1 h at roon tenperature. Cells were washed and resuspended in FACS buffer before analyzing then with flow cytonetry (CytoFLEX LX, Becknan Coulter; LSR-ll; B.D. Biosciences). Data analysis was done using the FlowJo software version 10.7.1 (BD Biosciences).

#### PD1/PDL1 Inhibition:

Human HEK293-K9PD1 cell line cells were blocked for 1 hr at room temperature using blocking buffer (FACS + 10% nornal nouse serun; lnnunoReagents cat#SP-002-VX5). Cells were labelled with prinary antibody (purified anti-PD1 Ab fron each cell line and NCF cells) for 1 hr at roon tenperature post-blocking. Unlabeled cells were used as a negative control. Cells were washed with FACS buffer (1X PBS + 1% B.S.A.; bovine serun albunin; VWR cat #0332, + 200nM EDTA, Therno ref #15575–038) and labelled with Zenon AF647 (Therno ref #Z25408) labeled canine PDL1 protein conjugated with hunan Fc (Therno: cat #P21462 ) for 1 hr at roon tenperature. Cells were washed and resuspended in FACS buffer before analyzing via flow cytonetry (CytoFLEX LX, Becknan Coulter; LSR-ll; B.D. Biosciences). Data analysis was done using the FlowJo software version 10.7.1 (BD Biosciences).

### Statistical analysis:

Statistical analysis was perforned using Python and Graph Pad. Linear regression was used to evaluate the relationship between viral dose (MOl) and cell viability in the 2D luciferase assay, with log-transforned MOl as the independent variable. Two-way ANOVA was applied to spheroid size data to assess the effects of tine, MOl, and their interaction on growth for each cell line separately. For tunor volune data, linear regression was used to e:anine changes over tine and to evaluate the interaction between tine and treatnent (virus-treated vs. PBS).

## Results:

### Cytotoxic and cytopathic effects of CAV2-AU-M3:

#### 2D:

Four canine osteosarcoma cell lines, D17-GLP, CF11-GLL, D22-GLP, and MCKOS that express GFP/ffluc were used to test the inter-line tumor heterogeneity. Cytotoxicity was observed using the Luciferase cytotoxicity assay (cell viability assay). There were no significant differences in cell viability at 0, 10, and 100 MOI in D22-GLP and MCKOS cell lines on day 5, but some cytotoxicity was evident (rapid cell rounding and progression to conplete cell death) in D17-GLP and CF11-GLL (Figure 1B; S1–12). However, we observed that at MOIs of 1000 or higher, CAV2-AU-M3 lysed all cell lines efficiently by day 5, but at varying percentages (Figure 1D). D17-GLL and CF11-GLL cell lines showed clear cell lysis at MOls greater than 500 (Figure AD; S1–12). D17 GLP and CF11 GLL cell lines have higher as well as TCID50 (Figure 1G) values, also in comparison to MCKOS and D22 GLP cell lines. CAV2-AU-M3 showed CPE in MCKOS and D22-GLP cell lines at MOls above 5000, conpared to the negative control (Figure 1D). Linear regression analysis using log-transformed MOI demonstrated a significant negative association between viral dose and cell viability across all cell lines (p < 0.001). Increasing MOI was associated with a reduction in viability in D17-GLP (β = −21.40, p < 0.001), CF11-GLL (β = −22.22, p < 0.001), D22-GLP (β = −8.48, p < 0.001), and MCKOS (β = −14.40, p < 0.001). CF11-GLL and D17-GLP cell lines exhibited the steepest declines in viability, indicating higher sensitivity to increasing viral dose. In contrast, D22-GLP showed a more gradual reduction, suggesting relative resistance, while MCKOS demonstrated an intermediate response. Evidently, CAV2-AU-M3 produces more CAV2 virus particles in D17-GLP and CF11-GLL in comparison to MCKOS and NCF cells (Figure 1C). D17-GLP and CF11-GLL cell lines had the highest TCID50/ml (1.26 X 10^5^) in comparison to MCKOS (1.26 X 10^4^) and D22-GLP (1.26 X 10^3^) (Figures 1E, F, & G).

#### 3D:

##### Spheroid growth and cell lysis:

**Figure 2:**
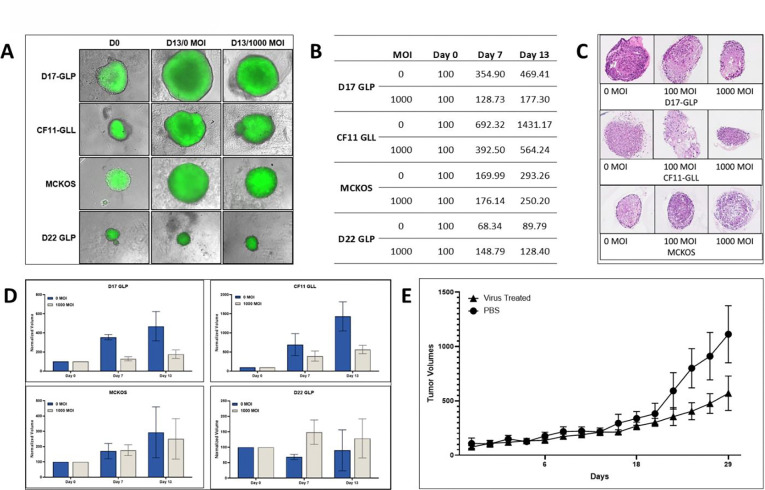
CAV2-AU-M3’s infectious and cytolytic properties in canine osteosarcoma cell lines, spheroids, and a xenograft murine model. (A) OS spheroids were infected with 1000 MOIs of CAV2-AU-M3, and spheroid size was visualized using inverted fluorescent microscopy (Keyence) after 13 days of infection. (B) Spheroid size on days 0, 7, and 13 at 0 and 1000 MOIs. (C) Histological sections of D17GLP, CF11GLL, and MCKOS spheroids, infected with 0, 100, and 1000 MOIs after 13 days of infection. (D) Spheroid volumes as a percentage of the same OS cell line at day 0 shown at days 0, 7, and 13, at 0 and 1000 MOIs. (E) Xenograft tumor volumes in PBS and CAV2-AU-M3-treated groups. Tumors were monitored up to 30 days.

D17-GLP, CF11-GLL, and MCKOS spheroids were infected at 0, 100 (S13–23), and 1000 MOIs of CAV2-AU-M3 for 13 days. All four cell lines responded differently to CAV2-AU-M3 infections in 3D. A 2-way ANOVA incorporating time, MOI, and their interaction revealed heterogeneous growth responses across the osteosarcoma cell lines. There were no or minimal changes in spheroid volume between 0 and 1000 MOIs in D22-GLP and MCKOS spheroids (Figures 2A, B, & D), even at day 13. The MCKOS cell line showed a significant increase in size over time (β = 14.73, p = 0.017), with no significant interaction between time and MOI (p = 0.695 for 1000 MOI). D22-GLP cell line spheroids exhibited no significant change in size over time (β = −0.89, p = 0.706), and no significant effects of MOI or interaction were detected (p > 0.05), suggesting minimal responsiveness to treatment.

In the D17-GLP cell line, the infected spheroids were bigger in size in comparison to day 0, but spheroids infected with CAV2-AU-M3 had less growth in comparison to non-infected spheroids. There is a significant difference in spheroid volume between 0 and 1000 MOIs of infections (Figures 2A, B, & D) on day 7 (p = 0.0005) and almost significant on day 13 (p = .0711). CF11-GLL spheroids were significantly smaller in comparison to non-infected spheroids both on day 7 and 13 at 1000 MOIs (Figures 2A, B, & D). CF11-GLL cell line cells demonstrated a strong time-dependent increase in spheroid size (β = 101.91, p < 0.001) and a significant negative interaction between time and 1000 MOI (β = −66.03, p = 0.001), indicating that high viral dose significantly suppressed spheroid growth over time compared to control. These findings indicate that, while spheroid growth increased over time in most cell lines, only D17-GLP and CF11-GLL showed a statistically significant reduction in growth rate with increasing MOI, highlighting cell line–specific sensitivity to viral treatment.

##### Spheroid histology:

Infected (100 and 1000 MOIs) and non-infected spheroids were collected on day 13. Spheroids were processed following the protocol described in the methods section, sectioned (4um), and stained with henato:ylin and eosin phlo:ine B stain. At 0 MOI, no tumor cell death was observed in D17-GLP, CF11GLL, or MCKOS cells. D17-GLP and CF11-GLL spheroids infected with CAV2-AU-M3 at 100 or 1000 MOI showed extensive tumor cell lysis. Tumor cell lysis is evident by cell shrinkage, loss of plasma membrane, cytoplasmic eosinophilia, and karyolitic or pyknotic nuclei. However, MCKOS spheroids were relatively resistant to CAV2-AU-M3-induced tumor lysis. (Figure 2C).

#### Animal model:

We tested the cytolytic effects of CAV2-AU-M3 in a murine xenograft osteosarcoma model in NOD.Cg-Prkdc^scid^ Il2rg^tm1Wjl^/SzJ mice. 5 million D17-GLP cells were injected subcutaneously into the left flank of 10 mice. A single intratumoral injection of 1 X 10^9^ VPs was performed when the tumor size reached 100 mm^3^ in 5 mice. 5 control mice were injected with 1X PBS. Tumor size was measured every alternate day for 30 days in both the control and treatment groups. Both groups had constant tumor growth in all mice. However, mice treated with CAV2-AU-M3 had slower growth in comparison to the control group, as seen in the spheroid model (Figure 2E). Linear regression analysis demonstrated a significant increase in tumor volume over time in the control (PBS) group (β = 30.73, p < 0.001). Importantly, a significant interaction between time and treatment was detected (β = −15.98, p < 0.001), indicating that tumor growth over time was significantly reduced in the virus-treated group compared to PBS. Specifically, the estimated growth rate in the virus-treated group (~14.75 units/day) was substantially lower than in the control group (~30.73 units/day), demonstrating that viral treatment effectively suppresses tumor progression.

### Characterization of His-Tagged Anti-PD1 Ab Produced and secreted by OS cells infected by CAV2-AU-M3

#### Anti-PD1 Ab size, production, and secretion verification:

**Figure 3:**
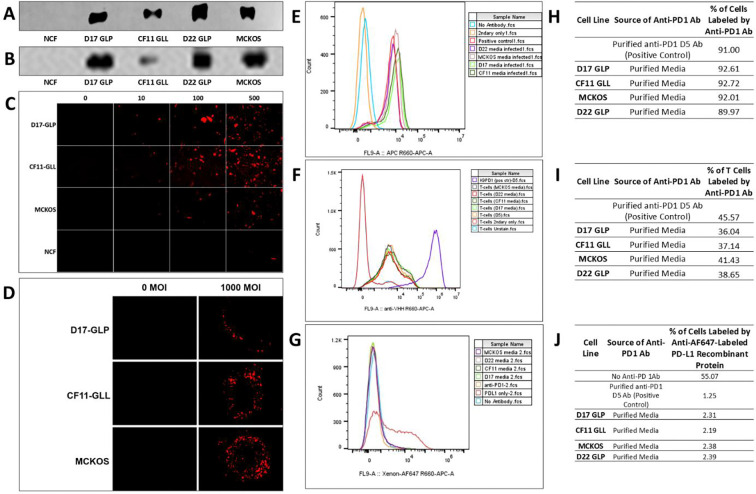
Functional properties of anti-PD1 HcAb isolated from cell culture media of D17GLP, CF11GLL, D22GLP, and MCKOS cell lines and NCF cells 72 hours after CAV2-AU-M3 infection at 100MOI. (A) Western blot analysis. Anti-PD-1 HcAb protein levels (monomers) were measured using mouse anti-6x-His (Thermo) primary antibody and IRDye 800 CW goat anti-mouse IgG (LI-COR) secondary antibody, and (B) Rabbit-anti-VHH-HRP primary antibody (genscript) LI-COR. (C) Immunofluorescence (Keyence; 10X) demonstrating the presence of anti-PD1 HcAb in monolayer cultures was performed in D17 GLP, CF11 GLL, and MCKOS cell lines, and primary NCF cells, at 0, 10, 100, and 500 MOIs (CAV2-AU-M3 infections for 48 hrs) using rabbit anti-VHH-iFluor 647 (GenScript) prinary antibody. (D) Immunofluorescence (Keyence; 10X) denonstrating the presence of anti-PD1 HcAb using the rabbit anti-VHH-iFluor 647 (GenScript) primary antibody was performed in D17 GLP, CF11 GLL, and MCKOS spheroids at 0 and 1000 MOIs after 13 days of infection. Images were captured using a Keyence 48 hrs post-infection. (E) Flow cytometry analysis of anti-PD-1 HcAb binding to PD-1 receptor on HEK293-K9 PD1 cells, with cell count on the y-axis and fluorescence on the x-axis. The binding of anti-PD-1 HcAb purified from cell media collected from osteosarcoma cell lines (D17 GLP, CF11 GLL, MC-KOS, and D22 GLP) infected with CAV2-AU-M3 was evaluated. (F) Flow cytometry analysis of anti-PD-1 HcAb binding to PD-1 receptor on T cells, with cell count on the y-axis and fluorescence on the x-axis. The binding of anti-PD-1 HcAb purified from cell media collected from osteosarcoma cell lines (D17 GLP, CF11 GLL, MC-KOS, and D22 GLP) infected with CAV2-AU-M3 was evaluated. (G) Flow cytometry analysis of PD-1/PD-L1 binding inhibition by anti-PD-1 HcAb. Inhibition of the binding of PD-L1 to PD-1 by anti-PD-1 HcAb purified from cell media collected from osteosarcoma cell lines (D17 GLP, CF11 GLL, MC-KOS, and D22 GLP) infected with CAV2-AU-M3. (H) Percentage population of HEK293 K9PD1 cells labeled by anti-PD1 HcAb isolated from OS cell lines. This table is the average of three independent experiments. (I) Percentage population of Canine T cells labeled by anti-PD1 HcAb isolated from OS cell lines. This table is the representation of three independent experiments. (J) Percentage population of HEK293 K9PD1 cells labeled by xenon AF647 labeled PDL1 protein due to anti-PD1 HcAb inhibition. This table is the average of three independent experiments.

Western blot was performed to verify the correct size, production, and secretion of His-tagged anti-PD1 Ab from CAV2-AU-M3 infected OS cells (Figure 3A & B). The secreted heavy chain antibody (HcAb) was labelled with anti-His-tag (Figure 3A) and an antibody against camelid VHH (Figure 3B) for verification. All four OSA cell lines (D17-GLP, CF11-GLL, MCKOS, and D22-GLP) produced and secreted anti-PD1 Ab in cell culture media. Both monomer (~42KD) and dimers (~84KD) were observed (Figure 3A & B; S24). We observed that the amount of anti-PD1 Ab was higher in the media than cell lysates across all four OS cell lines (Figure S24). There was no visible monomer anti-PD1 Ab secreted by primary NCF (normal canine fibroblasts) cells; however, NCF produced very low levels of anti-PD1 Ab dimers in comparison to OSA cell lines (Figure 3A & B; S24).

#### Immunofluorescence analysis of anti-PD1 secretion from OS cells:

OSA cell lines (D17-GLP, CF11-GLL, and MCKOS) and NCF were infected with CAV2-AU-M3 for 48 hours in monolayers before being stained anti-camelid VHH antibodies. All three OS cell lines showed anti-PD1 Ab production, as evidenced by the red fluorescence (Figure 3C). There was no or minimal production of anti-PD1 Ab in NCF cells (Figure 3C). Similarly, all three OS cell lines showed anti-PD1 Ab secretion in spheroids after 13 days of CAV2-AU-M3 infection (Figure 3D).

#### Confirmation of secreted anti-PD1 Ab binding with its ligand:

##### PD1+ HEK2G3-KGPD1 cells:

Flow cytometry was performed to analyze the binding capacity of anti-PD1 Ab secreted by CAV2-AU-M3-infected OS cells to its PD1 ligand. Anti-PD1 antibody released from all four infected cell lines demonstrated similar binding to PD1 cell surface receptor. A shift in the fluorescence in the histogram to the right shows anti-PD1 Ab binding canine PD1 surface receptor in HEK293-K9PD1 cells (Figure 3E & H). D17-GLP, CF11 -GLL, D22-GLP, and MCKOS media samples have shown binding to 91 %, 92.61 %, 89.97 % and 92.01 % respective cell population of HEK293-K9PD1 cells expressing canine PD1 ligand on the cell surface (Figure 3E & H).

##### T cells:

T cells isolated and purified from peripheral blood from a donor dog were activated using costimulatory antibodies (anti-CD3 and anti-CD28) and cultured for 10 days before analysis. anti-PD1 HcAb samples from all 4 infected cell lines (D17-GLP, CF11-GLL, D22-GLP, and MCKOS media) showed binding to canine T cells (Figure 3F & I).

#### Assessment of the Inhibition of PDL1 Binding to PD1:

##### HEK2G3-KGPD1 cells:

The functionality of anti-PD1 Ab secreted by CAV2-AU-M3 infected OS cells was assayed for its ability to block the PDL1 protein binding to PD1 receptor. HEK293 cells expressing canine PD1 receptors were labelled with AF647 zenon labelled canine PDL1 protein with and without the presence of anti-PD1 Ab (CAV2-AU-M3) (Figure 3G). A shift in the fluorescence in the histogram to the right after PDL1 labelling reverts back to the left, below the threshold when blocked with anti-PD1 antibody, demonstrating inhibition of PDL1 binding with PD1 receptor (Figure 3G). 55.07 percent of the population of HEK293-K9PD1 cells were labelled by Zenon-tagged canine PDL1 protein. The percentage population of HEK293-K9PD1 cells decreased in the presence of D17-GLP, CF11-GLL, D22-GLP, and MCKOS media samples containing secreted anti-PD1 Ab from 55.07 to 2.31 %, 2.19 %, 2.39 %, and 2.38 %, respectively (Figure 3J).

## Discussion

Osteosarcoma is a highly metastatic tumor with no major changes in survival outcomes in patients over the last four decades (28, 29). There is a pressing need to explore new therapeutic approaches to treat osteosarcoma. Cancer immunotherapies, such as oncolytic viruses and immune checkpoint inhibitors, are a promising avenue for more patient-tailored and effective therapeutic responses (30). Conditionally replicative oncolytic virus (CRADs) immunotherapy is a work-in-progress that has shown promising results in some tumors, including osteosarcoma (31, 32). T-VEC is an FDA-approved oncolytic virus currently used to treat melanoma (33). CRADs infect and specifically lyse tumor cells (34) and induce systemic anti-viral immunity along with anti-tumor immunity (35). Additionally, they can activate innate immunity, thereby triggering adaptive immunity in the TME (18).

We have developed a conditionally replicative oncolytic virus, CAV2-AU-M3, which is armed with an immune checkpoint inhibitor, anti-PD1 HcAb (Figure 1A). ICOCAV15 (36) was genetically modified to insert a secretory canine hinge and an Fc-conjugated camelid anti-canine PD1 single domain antibody between the fiber and E4 to produce CAV2-AU-M3. The resulting virus is ~107% the size of the CAV2 Toronto strain. Our hypothesis was that an armed oncolytic virus will be able to lyse cancer cells as well as produce and secrete the anti-PD1 HcAb payload in the tumor microenvironment. We demonstrated the cytolytic activity of CAV2-AU-M3 in four different osteosarcoma cell lines and its ability to produce and secrete a functional anti-PD1 antibody.

Virus-induced cell lysis results in visible cytopathic effects (CPE), such as cell rounding, detachment, and syncytia in a cell monolayer in cell culture (37). We observed that at a low multiplicity of infection (MOI) of 100, there was a visible CPE in D17-GLP and CF11-GLL; however, no such effect was observed in D22-GLP and MCKOS cell lines (Figure 1B). Immunofluorescence in OS cell lines and primary canine fibroblast cells (NCF) showed that the D17-GLP and CF11-GLL cell lines produced more CAV2-AU-M3 virus particles than MCKOS and NCF cells (Figure 1C). All four cell lines, when infected at higher MOIs of 500–10,000, showed varying levels of cell lysis (as measured by luciferase assay luminescence). D17-GLP and CF11-GLL showed significant cell lysis at 500 MOI and nearly 90% killing at 10,000 MOI after 5 days. MCKOS cells began showing significant cell lysis at 5,000 MOI, reaching nearly 70% at 10,000 MOI. D22-GLP cells also showed significant cell lysis at 5,000 MOI; however, unlike MCKOS, at 10,000 MOI, they showed only 40% cell lysis. We have observed that CAV2-AU-M3-induced cell lysis is more robust in fast-growing cell lines such as D17-GLP and CF11-GLL. Our data also demonstrated that CAV2-AU-M3 packages and lyses at different rates in different cell lines. TCID50 values of CAV2-AU-M3 in the MCKOS cell line are 10-fold more than D17 GLP and CF11-GLL (Figures 1D, E, & F). Similarly, the TCID50 value in D22-GLP is 10-fold lower than in MCKOS cell lines. Hence, CAV2-AU-M3 can infect and lyse tumor cells in monolayer cell cultures; however, at different ratios in different cell lines. The differences in cell lysis across cell lines could result from inter-individual heterogeneity.

Further, we cultured spheroids from all four OS cell lines to demonstrate the effect of CAV2-AU-M3 on spheroid growth. Tumor spheroids provide the 3D architectural features of solid tumors that are absent in 2D monolayers, making them a better model for studying viral penetration, replication, and therapeutic efficiency (38). We observed that the D17-GLP and CF11-GLL cell lines’ spheroids infected with 1000 MOI of CAV2-AU-M3 had a reduced growth over the course of time (13 days) compared to uninfected/0 MOI spheroids (Figure 2A). There was a significant size difference between infected and uninfected spheroids at days 7 and 13 in D17-GLP cell lines. The size difference is significant only on day 13 in CF11-GLP cell lines. However, no significant reduction in growth was observed in the D22-GLP and MCKOS cell lines (Figures 2B & 2D). As in 2D cultures, the virus effect was different across cell lines in 3D cultures as well.

Lastly, we demonstrated the effect of CAV2-AU-M3 in D17-GLP xenograft tumors in immunocompromised mice (Figure 2E). CAV2-AU-M3 was injected once intratumorally after tumors reached the size of 100mm^3^. Tumors were measured every other day. A single injection of CAV2-AU-M3 did not clear the tumors completely, but reduced the tumor growth. We monitored tumor growth for up to 30 days. A significant difference in tumor size between the control and virus groups was observed starting on day 22.

Based on the spheroid and xenograft tunor data, to attain a profound effect, we likely need to administer multiple doses of the virus to achieve complete tumor remission. It should be noted that the immunoregulatory aspect of the virus has little to no effect in the immunosuppressed murine strain required for xenograft survival, and thus efficacy will likely be enhanced in an immunocompetent model.

The second aspect of our virus is its ability to produce and secrete anti-PD1 HcAb. CRADs can be engineered to express the immunomodulatory proteins that can shape the tumor microenvironment (TME) against the tumor (39–41). Immune checkpoint inhibitors (ICIs), such as antibodies against PD1 and PDL1 (42–46) can release the immune-inhibitory “brakes” on T cells to restore anti-tumor immunity (47, 48). However, systematic delivery of ICIs can lead to off-target side effects and autoimmunity (49). Therefore, we designed next-generation CAV2-AU-M3, which will produce sustained levels of anti-PD1 Ab directly in the TME upon infection.

CAV2-AU-M3 is an enhanced version of our previously published CAV2-AU-M2 oncolytic virus (50). CAV2-AU-M3 produces secretory chimeric anti-PD1 Ab (Figure 1A). The anti-PD1 Ab comprises anti-canine camelid VHH and hinge and Fc domain of canine IgG D. The chimeric version of anti-PD1 Ab is bigger in size than nanobodies and has a higher half-life in TME (51). CAV2-AU-M3 is designed to produce and secrete anti-PD1 Ab in the TME upon tunor cell infection, along with viral replication, which is inportant for enhanced T cell function in the TME (52).

We infected all four cell lines with CAV2-AU-M3 and analyzed the production and secretion of anti-PD1 Ab in cell culture media. The anti-PD1 Ab produced makes monomers of ~42KD monomers (Figure 3A&B) and dimers of ~84KD (Supplementary D3). We observed that MCKOS and D22-GLP cell lines, although not sensitive to CAV2-AU-M3 cell lysis, did produce more anti-PD1 Ab at a lower MOI of 500 in comparison to D17-GLP and CF11-GLL(Figures 3A & B). D22-GLP showed similar cell lysis to NCF (TCID50; Figures 1E & F). However, NCF cells produce a lower level of anti-PD1 antibody than OS cell lines. There was no visible monomer band in NCF cells’ media (Figures 3A & B) and a minimal dimer band at the same MOI in comparison to all OS cell lines. This data demonstrates that CAV2-AU-M3 may be impaired in lysing some cell lines (D22-GLP and MCKOS compared to D17-GLP and CF11-GLL) but can still produce anti-PD1 Ab at similar levels.

To further test this hypothesis, we perforned innunofluorescence against anti-PD1 VHH in both 2D and 3D cultures. Conpared with Figure 1B, it is reasonable to assune that although CAV2-AU-M3 does not produce and package virus particles equally across all OS cell lines, its genone is replicating and thus producing a conparable anount of anti-PD1 in low-packaging cell lines (MCKOS and D22-GLP; Figures 3B & C).

lCls, such as anti-PD1 antibodies, conpetitively inhibit the PD1-PDL1 interaction, thereby reversing T cell e:haustion in the TME and facilitating T cell killing of tumor cells (53, 54). It is not possible to demonstrate the effect of the enhanced CAV2-AU-M3 virus on the host’s immune response *in vitro*. However, we were able to demonstrate that anti-PD1 Ab produced by OS cell lines can bind to its intended receptor, PD1, and can also inhibit PD1 and PDL1 interaction (Figures 3E–H). As is evident in Figures 3F and I, anti-PD1 secreted by all OS cell lines binds the PD1 receptor expressed on HEK293 K9PD1 cells and on T cells isolated and activated from a healthy dog donor.

In addition to efficient PD1 binding, anti-PD1 Ab produced by CAV2-AU-M3 can effectively inhibit the PD1-PDL1 interaction (Figures G & J). Thus, CAV2-AU-M3 will be able to reverse T cell exhaustion in the TME upon infection and facilitate the stimulation of innate and adaptive immune responses against osteosarcoma.

Based on the data presented, we can deduce that CAV2-AU-M3 can infect and produce anti-PD1 Ab in multiple OS cell lines. It is apparent that CAV2-AU-M3 has a slight packaging disadvantage in slow-growing cell lines MCKOS and D22-GLP, but it can still efficiently produce anti-PD1 in cell culture media. This may be due to the viral genome replicating in the presence of free E2F in immortal cell lines, so nore copies are available to produce more anti-PD1 Ab. Production of anti-PD1 Ab is under the CMV pronoter in CAV2-AU-M3, and the nore gene copies, the more protein is produced. The proliferative cells have a high capacity for protein synthesis, which viruses can e:ploit to produce capsid proteins (55). Therefore, CAV2-AU-M3 packaging nay be lower in D22-GLP and MCKOS due to their low proliferation rates.

In conclusion, CAV2-AU-M3 will be able to infect, lyse, produce, and secrete anti-PD1 Ab in the host TME upon infection. However, in sone slow-growing tunors, CAV2-AU-M3 nay be inefficient in lysing tunor cells such as MCKOS and D22-GLP, however it can still initiate and enhance an innune response in the TME against the tunor by producing a functional anti-PD1 antibody. While we were able to produce an oncolytic virus with an immunostimulatory payload, further improvements are still needed for a better lytic oncolytic virus.

## Supplementary Material

Supplementary Files

This is a list of supplementary files associated with this preprint. Click to download.

• SupplementaryDataKhan2026.pdf

• Figures.pdf

## Data Availability

All data generated and analyzed during this study are included in the article and its Supplenentary Materials. Further supporting data is available fron the corresponding author upon reasonable request.
